# A Case of Transient Unilateral Right Leg Edema Caused by a Markedly Distended Bladder

**DOI:** 10.7759/cureus.42914

**Published:** 2023-08-03

**Authors:** Nobuhito Yagi, Kazuhito Hirata, Wataru Higashiura, Hiroaki Takara, Minoru Wake

**Affiliations:** 1 Cardiology, Okinawa Chubu Hospital, Uruma, JPN; 2 Radiology, Okinawa Chubu Hospital, Uruma, JPN

**Keywords:** intravascular ultrasound, deep vein thrombosis, distended bladder, neurogenic bladder, unilateral leg edema

## Abstract

External compression of a vein is a relatively rare but important cause of unilateral leg edema. Here, we present a case of unilateral right leg edema caused by external compression of the right iliac vein due to a markedly distended urinary bladder, secondary to a neurogenic bladder. The patient initially had bilateral leg edema associated with chronic heart failure. However, the right-leg edema worsened and remained painful for several days. Lower extremity ultrasonography and computed tomography revealed an enlarged bladder. Based on these findings, venous angiography and intravascular ultrasound were performed. External compression is a significant cause of leg edema. It is important to consider the possibility of intra-abdominal/pelvic processes that may lead to external compression of the venous system in patients with unilateral and even bilateral lower extremity swelling.

## Introduction

External compression of a vein is a relatively rare but important cause of unilateral leg edema. However, for patients presenting with bilateral leg swelling, a typical assessment involves the consideration of multiple diagnoses, including congestive heart failure, renal or liver disease, edema-inducing medications, bilateral chronic venous insufficiency, and bilateral deep vein thrombosis (DVT) or thrombosis of the inferior vena cava. In contrast, unilateral leg swelling may be caused by DVT, a ruptured popliteal cyst, cellulitis, erythema nodosum, and trauma. Pain is usually due to an inflammatory process, as seen in cellulitis, venous thrombosis, trauma, or ruptured Baker’s cyst [1].

Extrinsic venous compression can be caused by several pathologies, including abdominal or pelvic tumors, aortic aneurysm, retroperitoneal hematoma, or retroperitoneal fibrosis. Compression of the pelvic veins and inferior vena cava by a gravid uterus is common in the late stages of pregnancy, particularly in patients in the supine position [2].

Here, we present a case of unilateral right leg edema caused by external compression of the right iliac vein due to a markedly distended urinary bladder, secondary to a neurogenic bladder.

## Case presentation

An 81-year-old female developed right lower leg swelling and pain in the lower end of the right leg. The patient originally had bilateral leg edema associated with chronic heart failure; however, the edema of the right lower extremity worsened and became painful for several days. 

The patient had undergone both mitral and aortic valve replacement for rheumatic mitral and aortic valve stenosis 37 years ago. She also had chronic atrial fibrillation with atrioventricular block and ventricular tachycardia, for which she had received an implantable cardioverter-defibrillator nine years prior. She had severe tricuspid regurgitation and frequently developed right-dominant heart failure, resulting in bilateral leg edema. She was hospitalized for recurrent right heart failure with bilateral leg edema, for which she was treated with intravenous furosemide 40 mg/day and carperitide (human natriuretic polypeptide) 0.025γ, oral tolvaptan (vasopressin receptor antagonist) 15 mg, and spironolactone 25 mg. After treatment, the bilateral leg edema improved. However, the patient developed edema in the right leg for several days, which was accompanied by decreased urine output.

**Figure 1:**
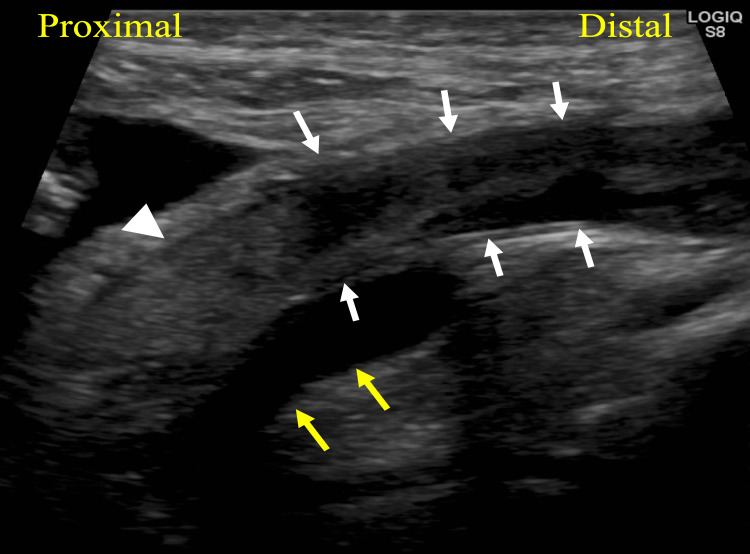
Venous ultrasonography. Lower extremity venous ultrasonography reveals a possible thrombus formation (white arrowhead), as manifested by heavy spontaneous echo contrast (white arrow) distal to the obstruction. White arrows delineate the right external iliac vein and yellow arrows indicate the right external iliac artery.

Lower extremity venous ultrasonography revealed a distended bladder compressing the right iliac vein and possible thrombus formation, as manifested by heavy spontaneous echo contrast distal to the obstruction (Figure 1).

**Figure 2:**
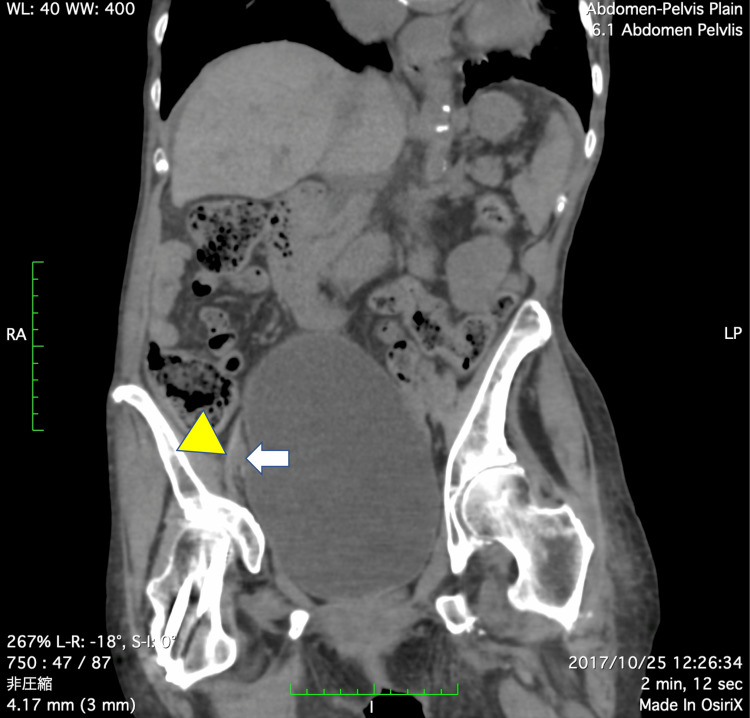
Computed tomography scan. Abdominal and pelvic computed tomography scans show the markedly distended bladder and right external iliac artery (white arrow) compressing the right external iliac vein (yellow triangle).

Abdominal and pelvic computed tomography (CT) images revealed a markedly distended bladder and severe scoliosis, but there were no tumors or enlarged lymph nodes (Figure 2). The severe scoliosis caused spinal cord injury and a neurogenic bladder. The patient tended to take a bending position on her right side for relief of back pain due to scoliosis. Based on these findings, we decided to perform venous angiography as a possible catheter intervention for venous obstruction.

**Figure 3:**
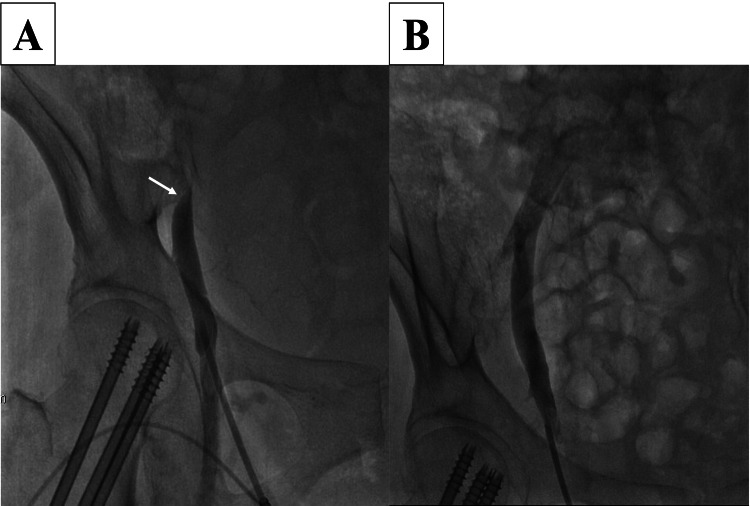
Venography. A: Venous angiography reveals complete obstruction at the right external iliac vein (white arrow). B: Venography showing normal flow without residual obstruction or stenosis after urinary catheter insertion.

**Figure 4:**
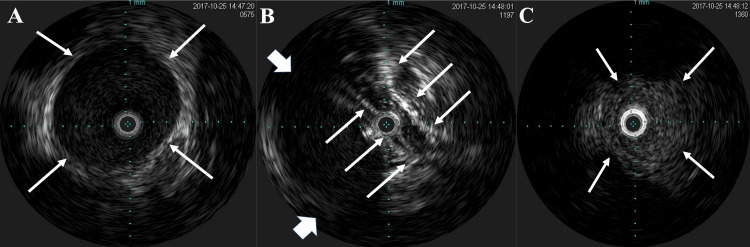
Intravascular ultrasonography. A: Intravascular ultrasonography showing normal structure of the common iliac vein proximal to the obstruction. White arrows delineate the wall of the common iliac vein. B: Intravascular ultrasonography image at the site of obstruction (white arrows) showing that the right external iliac vein was compressed and obstructed by the right external iliac artery (white arrowhead). C: Intravascular ultrasonography showing heavy spontaneous echo contrast distal to the obstruction, suggesting a thrombus. The white arrows indicate the wall of the right external iliac vein.

The right femoral vein was cannulated with a 4-French sheath, and venous angiography revealed complete obstruction of the right iliac vein (Figure 3, Panel A). An intravascular ultrasound (IVUS) catheter (Eagle Eye Platinum, Phillips) was advanced through the obstruction, which showed that the right external iliac vein was obstructed by the right external iliac artery and the distended bladder complex (Figure 4, Panels A, B). There was heavy spontaneous echo contrast distal to the obstruction, suggesting a thrombus (Figure 4, Panel C). After the sheath was changed to 7 French and the stenosis was penetrated with a Radifocus guidewire (0.035 in × 150 cm, Terumo Japan), and thrombus aspiration was attempted with an aspiration catheter (Vasplyser™ Plus, Cordis). However, the thrombus was not retrieved, the contrast medium that had been stagnant in the peripheral site flowed into the central part of the stenosis, and the obstruction was released by catheter insertion alone. The presence of an organic thrombus was ruled out.

When a urethral catheter was inserted into the bladder, 1,700 mL of urine was excreted. IVUS showed that the obstructed vein returned to its normal size, and venography showed normal flow without residual obstruction or stenosis (Figure 3, Panel B).

Finally, we diagnosed the patient with transient unilateral right-leg edema caused by a markedly distended bladder.

## Discussion

This report presented a case of unilateral right-leg edema caused due to external compression of the right iliac vein because of a markedly distended urinary bladder. In this particular case, the findings of an enlarged bladder on ultrasound and CT scan, in combination with angiographic and IVUS findings, established the diagnosis and treatment.

Venous obstruction resulting from a distended urinary bladder was first reported by Carlsson and Garsten in 1960 [3]. The most common cause of venous obstruction due to bladder distension was benign prostatic hypertrophy [4-6]. Presenting symptoms range from painless leg swelling to phlegmasia cerulea dolens and circulatory collapse.

At first, we thought that the decrease in urine output and the worsening of leg edema was due to the worsening of heart failure, but the symptoms did not improve even after heart failure treatment with intravenous furosemide and carperitide and dosing up of oral tolvaptan.

The patient suffered from severe scoliosis, which caused a spinal cord injury and a neurogenic bladder. She seldom expressed a desire to urinate and tended to take a bending position on her right side for relief of the back pain due to scoliosis. Hence, bladder fullness as the cause of abdominal distention was not obvious.

## Conclusions

External compression is a significant cause of leg edema. It is important to note and consider the possibility of intra-abdominal/pelvic processes that may lead to external compression of the venous system in patients with unilateral and even bilateral lower extremity swelling.
